# Emotion Regulation Difficulties and Academic Procrastination

**DOI:** 10.3389/fpsyg.2020.524588

**Published:** 2020-11-04

**Authors:** Jahangir Mohammadi Bytamar, Omid Saed, Sahel Khakpoor

**Affiliations:** Department of Clinical Psychology, Faculty of Medicine, Zanjan University of Medical Sciences, Zanjan, Iran

**Keywords:** academic procrastination, emotion regulation, difficulties in emotion regulation scale, anxiety, depression

## Abstract

**Objective and Background:**

Academic procrastination as deliberate postponement of academic tasks, despite being aware of its consequences, is a common phenomenon among students. Current conceptualizations of procrastination support the rule of emotion regulation difficulties in the psychopathology of this phenomenon. In this regard, the current study is aimed to investigate the role of difficulty in emotion regulation in academic procrastination.

**Method:**

The present study is a cross-sectional study. Participants were 250 students who completed Tuckman Procrastination Scale (TPS), and Difficulties in Emotion Regulation Scale (DERS).

**Result:**

Correlation analyses showed that the TPS has a significant positive association on overall DERS and all but one of the six dimensions (DERS-Awareness) of emotion regulation difficulties (*p* < 0.01). This association remained significant after controlling for anxiety and depression. Further, the multivariate regression showed that the only DERS dimension that could predict TPS was DERS-Strategies. Finally, individuals with a high level of procrastination reported greater DERS scores than those with a lower level.

**Discussion:**

Results indicate that difficulty in emotion regulation, especially the ones’ believe about his/her ability in regulating unpleasant emotions effectively, is important in procrastination. However, despite the limited association between DERS and TPS, the findings raise some potentially useful implications for procrastination studies and interventions.

## Introduction

Procrastination is defined as “to voluntarily delay an intended course of action despite expecting to be worse off for the delay” (Steel, 2010). It has various manifestations and forms such as General procrastination, Decisional Procrastination, Active Procrastination, Unintentional Procrastination, and Academic Procrastination. An overview of previous literature reveals that the most common form of procrastination is academic procrastination, which refers to the deliberate delay in completing academic assignments even though one is aware of its negative outcomes and consequences (Steel, 2007b). It has been reported that 15–20 percent of the adult and about half of the students suffer from chronic and frequent procrastination and suffer from major problems in their daily lives (Rozental and Carlbring, 2013). Research has also estimated that more than 60 percent of Iranian students involve in academic procrastination (Mohammadi Bytamar et al., 2018).

Academic procrastination is associated with major dysfunction at the academic performance (Madhan et al., 2012) and reduces psychological well-being (Van Eerde, 2003). Students who procrastinate are more likely to experience depression and social anxiety than students who don’t procrastinate (Mohammadi Bytamar et al., 2017). Procrastination is also associated with experiences of persistent stress and negative emotions, including anxiety, distress, depression, and hopelessness (Chabaud et al., 2010; Sirois, 2014).

Given that the procrastination is a multifactorial problem, different psychological, clinical, situational, and motivational factors have been proposed to explain it. This diversity in approaches leads the researchers to focus on more fundamental characteristics of procrastination such as self-regulation (Steel, 2007a; Kandemir, 2014). Self-regulation has been well studied with Self-Determination Theory (Ryan and Deci, 2017; Valenzuela et al., 2020). From this perspective, self-regulation is a motivational capacity that satisfies autonomous feelings and makes individuals pursuit and reach their personal goals. In this regard, procrastination can be consider as a volitional dysregulation, in spite of being motivated. This volitional problem can also effect on individual self-efficacy beliefs, motivation and goal settings through a vicious cycle (Valenzuela et al., 2020).

However, the more important question is why procrastinators fail in self-regulation. In attempt to answer this question, studies have identified range of factors, including factors related to task characteristics such as perceived aversiveness or difficulty of the task and the length of time it takes to receive a reward (Dewitte and Schouwenburg, 2002) as well as factors related to the individual’s characteristics such as high impulsivity (Rebetez et al., 2018), low conscientiousness (Watson, 2001), perfectionism (Rozental and Carlbring, 2014), and fear of failure (Haghbin et al., 2012). Some results suggest a connection between the self-regulation failure of procrastination and emotion regulation (Sirois and Pychyl, 2013).

Emotion regulation consists of different strategies to monitor and change the frequency, intensity, duration, emotional reactions, and the expression of a wide range of emotions, especially in the context of goal-related behavior (Kring, 2010; Gross, 2015). Adaptive emotion regulation strategies involve modulating the experience of intensive emotions in order to facilitate achieving desired goals (Gratz and Roemer, 2004). On the other hand, emotion dysregulation defined as “maladaptive emotional responsiveness reflected in dysfunctional understanding, reactivity, and management” (Mennin et al., 2007). Emotion regulation difficulties have been reported across a wide range of emotional problems (Berking and Wupperman, 2012; Naragon-Gainey et al., 2017; Khakpoor et al., 2019). Steel (2007a) considers procrastination as a kind of dysfunctional, emotional driven self-regulation. In his view, procrastinators consider themselves unable to change the situation, and thus, instead of focusing on the task, they turn their attention to their emotions and emotional responses. In other words, they choose the emotion-oriented style instead of the task-oriented style which resulted in postponing the aversive tasks.

Tice and Bratslavsky (2000) also suggest that difficulty in emotion regulation can be considered as one of the causes of failure in self-regulation and self-control. From this perspective, focusing on regulating emotions and prioritizing the short-term mood repair rather than pursuing goals in the long term, results in failure in self-regulation and therefore procrastination (Sirois and Pychyl, 2013). Evidence indicated that negative emotions are important antecedents of procrastination (Steel, 2007a; Wohl et al., 2010). When an individual confronts a task that is viewed as aversive, difficult, or boring, he/she experiences negative emotions (Solomon and Rothblum, 1984). In such circumstances, the procrastinator tries to get rid of these emotions as soon as possible by avoiding tasks or procrastination (Sirois and Pychyl, 2013). It seems that individuals postponing their tasks, due to lack of access to adaptive emotion regulation strategies and consequently failure in self-regulation (Fernie et al., 2016; Steel and Klingsieck, 2016) and by doing so, they at least temporarily avoid negative emotions (Golestanibakht and Shokri, 1392). However, the effect of procrastination on repairing mood is short term and does not have long-lasting effects (Sirois and Pychyl, 2013). Conversely, delaying goals or tasks imposes greater costs (such as time pressure) on the individual and caused more negative emotions such as guilt and anxiety and thus more procrastination, in the long-term (Fee and Tangney, 2000).

Although previous theories suggest emotion regulation as a predisposing and perpetuating factor in understanding procrastination (Sirois and Pychyl, 2013; Pychyl and Sirois, 2016), limited studies have examined the relationship between these two. These studies mainly concerned the procrastination intervention through enhancing emotion regulation skills (Mirzaei et al., 2014; Eckert et al., 2016) and not examined their connection specifically.

In this regard, the present study had four primary aims. The first goal was to examine the association between academic procrastination and an overall difficulty in emotion regulation and its dimensions, controlling for anxiety and depression. It was hypothesized that difficulty in emotion regulation and its dimensions could predict academic procrastination, beyond anxiety and depression levels. Second, the study aimed to investigate the incremental contribution of an overall difficulty in emotion regulation beyond anxiety and depression. It was hypothesized that difficulty in emotion regulation provide significant unique variance in predicting academic procrastination. Third, the current study aimed to determine which dimensions of difficulty in emotion regulation is particularly relevant to academic procrastination. It was hypothesized that limited access to emotion regulation strategies would probably be the best predictor of procrastination. However, according to the priority of short-term mood regulation, it seemed that in the second place, difficulty to perform purposeful behaviors related to emotions to be the next predictors. Finally, we sought to examine whether there is a different between individuals with high and low level of procrastination in reporting difficulties in emotion regulation as well as group differences in its dimension. It was hypothesized that individuals who were above the procrastination cutoff report more difficulties in emotion regulation than those below the cutoff.

## Materials and Methods

### Participants and Procedure

The participants consisted of 250 students recruited from the Zanjan University of Medical Sciences via a convenience sampling method. The participants were asked to complete all the scales including Beck Depression Inventory (BDI-II), Beck Anxiety Inventory (BAI), Tuckman Procrastination Scale (TPS), and Difficulties in Emotion Regulation Scale (DERS).

The inclusion criteria included age 18 or older, fluency in Persian, and the willingness to participate in the study. Given that psychological disorder(s) could contribute to the observed level of DERS, TPS, and the possible association between them, participations had been asked to report if they had been diagnosed with a psychological disorder(s) by a psychologist or psychiatrist. Eighteen participations reported psychological disorder(s) and excluded from the study. Further, 22 participations were excluded because they did not complete all the measures. The final sample consisted of 210 students with a mean age of 23.35 years (*SD* = 5.35). The sample contained 49% male and 51% female. In terms of education, 13.3% were associate students, 48.1% were undergraduate, 8.1% were postgraduate and 29.9% were Ph.D. students.

The study had been approved by the Ethics Committee of Zanjan University of Medical Sciences (reference number: ZUMS.REC.1396.79). All participations were submitted anonymously and written informed consent was obtained from them.

### Measures

#### Beck Depression Inventory (BDI-II)

This 21-item inventory was designed by Beck et al. (1996) to measure the severity of depression over the past 2 weeks. It has a high internal consistency (α = 0.91) and test-retest reliability of 0.93 in a week (Beck et al., 1996). The internal consistency coefficient for Iranian sample were 0.90 and the test–retest coefficient was 0.94 (Ghassemzadeh et al., 2005). In this study, Cronbach’s α was 0.86.

#### Beck Anxiety Inventory (BAI)

This 21-item inventory was designed by Beck et al. (1998) to measure the severity of anxiety in adults and adolescents. An adequate reliability value (Cronbach’s alpha coefficient of 0.93) and 5-week test-retest reliability coefficient (0.83) has been reported for this inventory (de Beurs et al., 1997). In Iran, an adequate internal consistency of BAI (α = 0.92) and test–retest (*r* = 0.83) has been reported (Rafiei and Seifi, 2013). In this study, Cronbach’s α was 0.93.

#### Difficulty in Emotion Regulation Scale (DERS)

This scale was developed by Gratz and Roemer in 2004 with 36 items and 6 subscales for measuring emotion dysregulation and emotional self-regulation strategies. The subscales of this scale include the lack of acceptance of emotional responses, difficulty in performing purposeful behavior, difficulty controlling impulse, lack of emotional awareness, limited access to emotion regulation strategies, and lack of clarity of emotion. High internal consistency (α = 0.93) and adequate 2-week test-retest reliability (*r* = 0.85) have been reported for this scale (Gratz and Roemer, 2004). The reliability of the Persian version of DERS also varies from 0.79 to 0.91, using the Cronbach’s alpha coefficient, and the reliability of its test-retest is between 0.86 and 0.88 after a week (Besharat and Bazzazian, 2015). Internal consistency in this sample was good with alphas ranging from 0.65 to 0.91 in six subscales and 0.91 in overall DERS.

### Tuckman Procrastination Scale (TPS)

This scale was developed by Tuckman (1991) to measure responders’ procrastination and to identify students with academic problems early in their career (Ferrari et al., 1995). The TPS consists of 16 Likert scale items and one factor. Twelve items are scored directly and four items (16-17-14-12) are scored inversely. Higher scores reflect greater procrastination tendency. Tuckman (1991) found good reliability with Cronbach’s alpha of 0.86. In Iran, an adequate internal consistency of TPS (α = 0.74) has been reported (Shehni et al., 2006). In this study, Cronbach’s α was 0.86.

### Data Analysis Method

SPSS version 25 was used to perform statistical analyses. Zero-order and the partial correlation coefficient was conducted to investigate the associations between DERS and TPS. Moreover, hierarchical and multivariate regressions were used to test the hypotheses that difficulty in emotion regulation and its dimensions would provide a significant contribution in predicting academic procrastination. Finally, univariate and multivariate analyses of variance (ANOVA/MANOVA) were calculated to compare DERS scores of individuals with a high level of procrastination to those at a subthreshold level. ANCOVA and MANCOVA were used to control for the effects of anxiety and depression.

## Results

Means, standard deviations, skewness, and kurtosis of all study variables are presented in Table 1. The Skewness and Kurtosis values of study variables showed that the distribution of variables is normal and in the range of −2 to +2. The Kolmogorov–Smirnov Test confirmed the assumption of normal distribution of study variables.

**TABLE 1:** Mean, Standard Deviation, Skewness, Kurtosis and normality test of study variables.

Variables	Mean	*SD*	Skewness		Kurtosis		Kolmogorov–smirnov test	
			Statistic	Standard error	Statistic	Standard error	Statistic	*p*-value
TPS total score	35.98	8.76	0.392	0.168	–0.360	0.334	0.163	0.695
Anxiety (BAI)	16.65	12.60	0.792	0.168	–0.198	0.334	0.211	0.380
Depression (BDI-II)	11.92	9.63	1.014	0.168	0.579	0.334	0.289	0.095
DERS total score	84.16	20.27	0.419	0.168	–0.426	0.334	0.176	0.607
DERS NON-ACCEPTANCE	12.80	5.04	0.491	0.168	–0.585	0.334	0.195	0.480
DERS GOALS	14.47	4.76	0.203	0.168	–0.602	0.334	0.187	0.529
DERS IMPULSE	14.10	5.16	0.678	0.168	–0.211	0.334	0.204	0.420
DERS AWARENESS	15.02	3.98	0.257	0.168	0.129	0.334	0.176	0.607
DERS STRATEGIES	17.64	6.73	0.646	0.168	–0.520	0.334	0.211	0.380
DERS CLARITY	10.13	3.44	0.888	0.168	0.708	0.334	0.218	0.434

*TPS, Tuckman Procrastination Scale; BAI, Beck Anxiety Inventory; BDI-II, Beck Depression Inventory-Second Edition; DERS, Difficulties in Emotion Regulation Scale; Non-accept, non-acceptance of emotional responses; GOALS, difficulty engaging in Goal-directed behavior; IMPULSE, impulse control difficulties; AWARENESS, lack of emotional awareness; STRATEGIES, limited access to emotion regulation strategies; CLARITY, lack of emotional clarity.*

No gender differences were found in tendency toward procrastination (M_men_ = 36.72, SD_men_ = 9.42; M_women_ =35.27, SD_women_ = 8.05; t=1.198, p=0.232) and total score of difficulties in emotion regulation (M_men_ = 85.84, SD_men_ = 21.06; M_women_ = 81.49, SD_women_ = 19.34; t=1.563, p=0.120). Regarding DERS subscales, women showed greater scores than men in lack of emotional awareness (M_men_ = 14.32, SD_men_ = 3.99; M_women_ = 15.70, SD_women_ = 3.86; t=−2.546, p=0.012) and men showed greater scores than women in impulse control difficulties (M_men_ = 15.28, SD_men_ = 5.70; M_women_ = 12.96, SD_women_ = 4.29; t=3.337, p=0.001) and limited access to emotion regulation strategies (M_men_ = 18.58, SD_men_ = 7.00; M_women_ = 16.74, SD_women_ = 6.36; t=1.999, p=0.047). Furthermore, no gender differences were found in non-acceptance of emotional response (M_men_ = 13.36, SD_men_ = 5.22; M_women_ = 12.26, SD_women_ = 4.83; t=1.582, p=0.115), difficulty engaging in goal-directed behavior (M_men_ = 14.82, SD_men_ = 5.06; M_women_ = 14.14, SD_women_ = 4.43; t=1.029, p=0.305) and lack of emotional clarities (M_men_ = 9.96, SD_men_ = 3.30; M_women_ = 10.30, SD_women_ = 3.57; t=−0.711, p=0.478). To explore the relationship between TPS and different DERS subscales, zero-order correlations were conducted (see Table 2). An examination of zero-order correlations showed that the procrastination (TPS) was significantly positively associated with DERS-total score, and all but one of the six dimensions of emotion regulation difficulties (*p* < 0.0001; Table 2). The DERS-Awareness was not significantly associated with TPS and the DERS-Strategies has the most powerful relationship with TPS. Partial correlations controlling for anxiety and depression were also computed to ensure that the above correlations could not be attributed to shared variance with these variables. TPS remained significantly associated with DERS-total and all of its dimensions except for the DERS-Awareness after controlling for anxiety and depression (see Table 2).

**TABLE 2:** Correlations between academic procrastination and difficulty in emotion regulation (controlling and not controlling for anxiety and depression).

Variables	1	2	3	4	5	6	7	8
(1) TPS total score	–	0.357**	0.255**	0.291**	0.294**	−0.015	0.358**	0.245**
		(0.266**)	(0.151*)	(0.193**)	(0.167*)	(0.001)	(0.217**)	(0.146*)
(2) DERS total score		–	0.722**	0.722**	0.811**	0.249**	0.847**	0.684**
(3) DERS NON-ACCEPTANCE			–	0.347**	0.461**	0.029	0.599**	0.367**
(4) DERS GOALS				–	0.722**	−0.037	0.522**	0.369**
(5) DERS IMPULSE					−	−0.031	0.624**	0.423**
(6) DERS AWARENESS						–	0.006	0.330**
(7) DERS STRATEGIES							–	0.510**
(8) DERS CLARITY								–
								

Results in parentheses represent partial correlations between variables when controlling for anxiety and depression. TPS, Tuckman Procrastination Scale; DERS, Difficulties in Emotion Regulation Scale; Non-acceptance, Non-acceptance of emotional responses; GOALS, difficulty engaging in Goal-directed behavior; IMPULSE, impulse control difficulties; AWARENESS, lack of emotional awareness; STRATEGIES, limited access to emotion regulation strategies; CLARITY, lack of emotional clarity. *p < 0.05, **p < 0.01.

Hierarchical multiple regression was conducted to examine the relative contributions of anxiety, depression, and difficulty in emotion regulation in predicting TPS more fully. DERS total score was used in this analysis as it remained significant predictors of TPS after controlling for anxiety and depression in the previous analysis. Anxiety was entered as a predictor variable at Step 1, followed by depression at Step 2. In the third and final step of the model, the DERS total score was entered. TPS served as the dependent variable. While anxiety accounted for a significant amount of variance in predicting TPS (3%) the addition of depression at step 2 accounted for an additional 10% of the variance; in fact, anxiety was no longer a significant predictor of TPS after the inclusion of depression in the model. Finally, when the DERS total score was entered in the third step, it significantly improved the model, accounting for an additional 7% of the variance in TPS and emerging as a significant predictor of TPS. BDI remained a significant predictor, although its beta weight decreased (see Table 3).

**TABLE 3:** Hierarchical regression analysis examining difficulties in emotion regulation as a predictor of TPS, controlling for BAI and BDI.

Variables		Beta	*R* (Adj. *R*^2^)	Δ*R*^2^	*F*
Step 1			0.18 (0.03)	0.03	6.73**
	Anxiety (BAI)	0.18**			
Step 2			0.38 (0.13)	0.10	16.90**
	Anxiety	–0.03			
	Depression (BDI-II)	0.38**			
Step 3			0.43 (0.17)	0.07	15.50**
	Anxiety	–0.06			
	Depression	0.29**			
	DERS total score	0.24**			

BAI, Beck Anxiety Inventory; BDI-II, Beck Depression Inventory-Second Edition; DERS, Difficulties in Emotion Regulation Scale. **p < 0.01.

In order to investigate the extent to which DERS dimensions predict procrastination in students, multivariate regression was conducted.

As seen in Table 4, the difficulty in emotion regulation dimensions could explain 15% of the total procrastination variance (*R*^2^ = 0.15). The results of one-way ANOVA showed that prediction of procrastination by dimensions of difficulty in emotion regulation was significant (*F* = 5.92, *p* < 0.01), which means that the relationship between procrastination and linear least-squares of the difficulty in emotion regulation dimensions cannot be random and the model is statistically significant. The results of multivariate regression also revealed that DERS-Strategies was the only DERS dimension that could significantly positively predict TPS (β = 0.220, *p* < 0.05). None of the other dimensions of DERS could significantly predict TPS.

**TABLE 4:** Regression analysis examining difficulties in emotion regulation subscales as a predictor of TPS.

Variables	Beta	*R* (*R*^2^)	Adj. *R*^2^	*F*
		0.39 (0.15)	0.12	5.92**
DERS NON-ACCEPTANCE	0.044			
DERS GOALS	0.116			
DERS IMPULSE	0.018			
DERS AWARENESS	−0.039			
DERS STRATEGIES	0.220*			
DERS CLARITY	0.079			

BAI, Beck Anxiety Inventory; BDI, Beck Depression Inventory; DERS, Difficulties in Emotion Regulation Scale. *p < 0.05, **p < 0.01.

Finally, a univariate analysis of variance (ANOVA) was conducted to compare DERS scores between individuals higher and lower than scoring 41 on the TPS. Individuals above the cutoff scored significantly higher on the DERS, *F*(1,208) = 12.13, *p* < 0.01 (see Table 5), with results remaining significant after controlling for anxiety and depression, *F*(1,206) = 3.89, *p* < 0.05.

**TABLE 5:** Differences in DERS total and subscale scores among participants above and below the TPS cutoff.

Variables	Above TPS cut-Off		Below TPS cut-Off		
	Mean	*SD*	Mean	*SD*	*F*
DERS total score	81.22	19.55	92.10	20.31	12.13**
DERS NON-ACCEPTANCE	12.23	4.73	14.33	5.56	7.45**
DERS GOALS	13.98	4.77	15.79	4.49	6.15*
DERS IMPULSE	13.59	5.12	15.46	5.04	5.53*
DERS AWARENESS	15.18	3.82	14.61	4.38	0.83
DERS STRATEGIES	16.48	6.41	20.77	6.23	18.30**
DERS CLARITY	9.76	3.14	11.14	4.00	6.89**

DERS, Difficulties in Emotion Regulation Scale; Non-accept, Non-acceptance of emotional responses; GOALS, difficulty engaging in Goal-directed behavior; IMPULSE, impulse control difficulties; AWARENESS, lack of emotional awareness; STRATEGIES, limited access to emotion regulation strategies; CLARITY, lack of emotional clarity. *p < 0.05, **p < 0.01.

Next, a multivariate analysis of variance (MANOVA) was conducted with the DERS dimension as dependent variables and the TPS variable (above vs. below the cutoff) as the independent variable. The overall model was significant, Wilkes λ = 0.91, *F*(6,208) = 3.48, *p* = 0.003. *Post hoc* ANOVAs demonstrated that above TPS cutoff participants reported significantly greater scores on all dimensions, except for DERS-Awareness (see Table 5). In a MANCOVA controlling for anxiety and depression, the overall model was no longer significant [Wilkes λ = 0.95, *F*(6,201) = 1.69, *p* = 0.125]. However, *post hoc* ANCOVAs revealed a significant difference in DERS-Strategies between the two groups, *F*(1,206) = 7.52, *p* = 0.07.

## Discussion

This study aimed to investigate the role of difficulty in emotion regulation and its dimensions in students’ academic procrastination. As expected, the overall DERS as well as its dimensions (except for DERS-Awareness) were positively significantly associated with academic procrastination, beyond shared variance of anxiety and depression. This finding is consistent with previous studies that support the role of difficulty in emotion regulation in procrastination (Rebetez et al., 2015; Zarei and Khoshouei, 2016) and suggests a unique association between TPS and the overall emotion regulation difficulties. Eckert et al. (2016) suggested that emotion regulation as a potential predictor of procrastination and that training of emotion regulation skills could reduce procrastination. This finding is also in line with theories that consider procrastination as a failure in self-regulation and maladaptive emotion regulation strategy (Steel, 2007a; Pychyl and Sirois, 2016). When procrastinators faced with tasks or situations that are seen as difficult or aversive, they prefer to regulate the negative emotions of the task immediately instead of pursuing their goals. For example, they try to reduce the experience of negative emotions through avoiding situations that provoke negative emotions, distracting themselves from aversive tasks, or biased information processing (Koole, 2009; Gross, 2015). In other words, rather than using adaptive emotion regulation strategies, they use procrastination as a way of regulating their emotions; a strategy that reduces negative emotions in the short term but prevents them from achieving their goals in the long term.

Surprisingly, the DERS-Awareness was negatively associated with procrastination at the bivariate level, although this relationship was not significant. There are some possible explanations for this finding. First, this finding suggests that the DERS-Awareness cannot be considered as a predicting factor for academic procrastination. In other words, people who procrastinate are probably as aware of their emotions as those who aren’t. Second, it appears that the DERS-Awareness measures a different construct from emotion regulation. The DERS is generally attempted to assess individuals’ *reaction* to unpleasant emotions, while this dimension focuses solely on the *awareness* of these emotions (Hallion et al., 2018). It is also worth to mention that emotional awareness has adaptive and maladaptive aspects which should be differentiated (Lischetzke and Eid, 2003). In this regard, the lack of association between DERS-Awareness and procrastination might be due to the inability of this dimension in distinguishing between two aspects of emotional awareness. It seems that procrastinators pay attention to their emotions in a maladaptive way when confronted with difficult tasks; which exposed them to the more unpleasant emotions and increased the chances of postponing assignments. Finally, this finding may be due to the psychometric limitations of the DERS-Awareness dimension. Studies which have been conducted on the psychometric properties of the DERS revealed that there is a weak association between the DERS-Awareness and other dimensions (Giromini et al., 2012; Fowler et al., 2014) and suggested to exclude its items from the scale (Osborne et al., 2017).

An examination of the relative contributions of anxiety, depression, and difficulty in emotion regulation in predicting procrastination indicated that DERS total score and DERS-Strategies were significant predictors of TPS and accounted for a significant incremental variance.

Overall, these findings suggest that how one regulates his/her emotion specifically, the *subjective appraisal* of the effectiveness of one’s efforts is an important factor in predicting an individual’s procrastination behaviors. DERS-Strategies measure “the belief that there is little that can be done to regulate emotions effectively, once an individual is upset” (Gratz and Roemer, 2004). In this regard, procrastination seems to be related to subjective appraisal about the ability to effectively modulate unpleasant emotions rather than using strategies. Procrastinators perceive themselves as someone who cannot change the situation effectively or regulate their negative emotions; the belief that makes them less likely to concentrate on the task at hand or tolerate unpleasant emotions to achieve their goals. It seems that this perception of self, along with the priority of short-term mood repair (Sirois and Pychyl, 2013), develops and perpetuates the procrastination behavior. This finding is especially important in clinical settings in which clinicians should address procrastinators’ appraisals by restructuring cognitions related to emotion regulation as part of procrastination interventions.

Moreover, previous literature conceptualized procrastination as a situation-dependent phenomenon (Patrzek et al., 2012; Klingsieck et al., 2013), a feature that has been considered in developing DERS-Strategies dimensions (Gratz and Roemer, 2004). It seems that the characteristics of a situation or goal can influence how individuals regulate their emotions and consequently their procrastination behavior. In other words, the inability to the flexible use of situationally adaptive emotion regulation strategies can hinder individuals from achieving goals or meeting environmental demands. For example, Codina et al. (2018) suggest that autonomy-supportive teaching style has a negative association with procrastination. It seems that this style of teaching can affect students’ procrastination through promoting motivation and regulation conditions.

Finally, finding indicated that individuals with a high level of procrastination report significantly greater level of overall difficulties in regulating their emotions. According to previous studies, the higher level of procrastination is associated with greater experience of negative emotion (whether as an antecedent or as a consequence) (Chabaud et al., 2010; Patrzek et al., 2012), which exacerbates the need to use strategies to recover their emotions. Result also indicated that DERS-Strategies was the only dimension which remained significant after controlling for anxiety and depression. Prior research emphasis on the role of self-efficacy (Ziegler and Opdenakker, 2018; Liu et al., 2020) and self-efficacy for self-regulation (Klassen et al., 2008) in predicting levels of procrastination. Self-efficacy can serves as a motivational, cognitive, affective and selective process in procrastination (Bandura, 1977). It improves individuals’ positive expectations, reduces negative emotions, and affects their efforts before and during doing the task. It seems that in the absence of anxiety and depression, individuals’ belief about their ability to influence the situation effectively (self-efficacy), has a remarkable role in procrastination severity.

The present study has some limitations. First, the sample size of the study was small, which makes it difficult to generalize the findings. In addition, the cross-sectional nature of the study did not allow an accurate understanding of the causal relationship between emotion regulation and procrastination. However, it seems that emotion regulation strategies, especially individuals’ perception of their ability in regulating emotions, can help formulate problems related to procrastination. Moreover, the current study only investigates difficulties in emotion regulation in explaining procrastination and doesn’t consider other underlying factors such as experiential avoidance (Heshmati et al., 2018), ruminations, and worry (Constantin et al., 2018), family cultural capital, habits, attitudes (Valenzuela and Codina, 2014) and teaching styles (Codina et al., 2018). Future studies would benefit from employing methods such as structural equation analysis or a longitudinal study design to investigate the causal relationship between these structures more accurately. Furthermore, it seems that despite the widespread use and high validity of the scale of DERS, this measure cannot fully assess emotion regulation in the context of procrastination. Some studies have suggested that there is an overlap between the content of items of DERS and structures such as attentional, planning, and decision-making difficulties (de la Cruz et al., 2013) which are important in describing procrastination. Some part of the association that has been found in the present study may be due to this overlap. In this regard, future studies should continue to investigate the exact relationship between emotion regulation and academic procrastination. It is also suggested future studies need to be done to develop and utilize a measure that can evaluate emotion regulation in this group of individuals. In addition, the main limitation of TPS is an interpretation based on the total score and it’s not address other dimensions of academic procrastination. Finally, the current study has evaluated the association between emotion regulation and procrastination solely on the conceptualization of Steel and Sirois. Although comparing different conceptualizations of emotion regulation (whether as a trait or a state) was not the aims of the present study, it is valuable to explore different emotion regulation models in future studies. These studies are especially important in helping us to expand our understanding of emotion regulation strategies in procrastination. The results of this study highlight the significant role of difficulties of emotion regulation in academic procrastination. Accordingly, the development and implementation of emotion-focused therapeutic interventions can help treat academic procrastination.

## Conclusion

The present study aimed to examine the role of difficulty in emotion regulation in the context of procrastination. Results revealed that procrastination is associated with difficulty in emotion regulation, especially individuals’ subjective appraisals about their ability to modify the situation. This finding is in line with models that consider emotion regulation as self-regulation failure in procrastination (Pychyl and Sirois, 2016).

The results of the present study raise some potentially useful clinical and academic implications. For example, individuals’ perceptions of the ability to effectively regulate their emotions can be considered as one of the therapeutic goals in procrastination interventions and academic consultations. Preliminary researches also indicated that interventions that increase flexibility in the use of emotion regulation strategies as their therapeutic goals can improve individuals’ procrastination (Glick et al., 2014; Gagnon et al., 2018). In this regard, further studies are needed to determine which dimension of difficulty in emotion regulation contributes to changes in procrastination during the interventions. These studies are important to expand the effectiveness of procrastination interventions.

## Data Availability Statement

The original contributions presented in the study are included in the article/supplementary material, further inquiries can be directed to the corresponding author.

## Ethics Statement

The studies involving human participants were reviewed and approved by Ethics Committee of Zanjan University of Medical Sciences. The patients/participants provided their written informed consent to participate in this study.

## Author Contributions

JM and OS carried out and designed the experiment. SK wrote the manuscript with support from OS. JM analyzed the data. All the authors discussed the results and commented on the manuscript.

## Conflict of Interest

The authors declare that the research was conducted in the absence of any commercial or financial relationships that could be construed as a potential conflict of interest.
